# Cause of Insanity

**Published:** 1913-11

**Authors:** 


### Cause of Insanity.

The Charities Aid Association of New York asks why should anyone go insane? They then answer the question in an interesting pamphlet for popular reading. They tell us that care of the insane cost New York State $54,000,000 in ten years and that the yearly expenditure for that purpose is about one-sixth of the entire cost of the State government. The annual cost for care of the insane equals a charge of 70 cents against each man, woman and child in the State. This for care. Practically none of it is for prevention.

These figures hold about true for each State in the Union. If the poulation of a State is multiplied by seven-tenths the result will be about the annual cost in dollars for the care of the insane.

In New York State in twenty years—1890 to 1910—the insane in hospitals increased twice as fast as the population. Here, too, the figures hold about true for the other States.

The report says: "Of all those admitted to the State hospitals one-fourth are cured and 80 per cent of these cures occur within a year after admission. The cure of insanity is well worthy of careful study. Each State must encourage the officers of its hospitals to attend meetings, read papers, study the literature and study their cases, for the insane are not a group for whom there is no hope.

Of greater importance to the public is the possibility of prevention. Justice Hughes of the Supreme Court says that "avoidable causes of insanity account for about 50 per cent of the patients under treatment."

First on the list of causes cited is immoral living. They say: "Over the door of every immoral resort might truthfully be written: 'Incurable insanity may be contracted here.' " In 1910, 17 per cent of the men and 8 per cent of the women admitted to New York asylums were insane from this group of causes.

Their next group is headed "Alcohol and Other Poisons." The next heading is "Physical Diseases." Under this head they say: "The control of infectious diseases, protection of food and water, temperance, healthful homes and factories—all these help to prevent mental as well as physical disease."

The next heading is "Mental Habits." Henry Ward Beecher once said: "It is not work that kills men; it is worry."

The last heading is "Heredity." The fact that heredity plays a part in the causation of insanity should create a public conscience regarding marriage.—*Chicago Tribune.*

